# Dynamic hand exercise recognition for game-based finger rehabilitation

**DOI:** 10.1038/s41598-026-42693-8

**Published:** 2026-03-12

**Authors:** Oladayo S. Ajani, Daison Darlan, Esther Aboyeji, Kalyana C. Veluvolu, Rammohan Mallipeddi

**Affiliations:** 1 School of Computer Science and Engineering, Kyungpook National University, Daehak-ro, 41566 Buk-gu, Daegu South Korea; 2 Department of Artificial Intelligence, Kyungpook National University, Daehak-ro, 41566 Buk-gu, Daegu South Korea; 3 School of Electronics Engineering, Kyungpook National University, Daehak-ro, 41566 Buk-gu Daegu, South Korea

**Keywords:** Hand gestures, Finger rehabilitation, Control, Transfer learning, Exergames, Therapeutics, Rehabilitation

## Abstract

Frequent and intense exercise is crucial for rehabilitation, but motivation is often a barrier. Recent studies indicate that exergames can enhance motivation and exercise intensity by using targeted motor function for game control. Generally, one way to achieve this is to enable the control of such games by the targeted motor function. For example, in finger rehabilitation, selected exercises control exergames, relying on both representative hand gestures and accurate recognition systems. However, existing recognition systems in the literature are modeled based on hand gesture datasets that are not representative of common finger rehabilitation exercises. Therefore, this work deviates from the pull of previous works by developing a hand-gesture recognition system using a dataset collected specifically for the purpose of finger rehabilitation. The dataset comprises RGB images collected from 14 different subjects while performing 7 different finger exercises under varying backgrounds and lighting conditions. A learning network that leverages transfer learning of an off-the-shelf pre-trained VGG16 model using both feature extraction and fine-tuning is developed to recognize the seven different hand gestures featured in the dataset. The resulting models achieved an accuracy of 82.38% and 85.12% before and after fine-tuning respectively. Furthermore, the misclassification rate observed for specific classes was analyzed using the class activation map. The resulting model is integrated into an exergame framework and an experimental study conducted with 15 unimpaired participants demonstrates the suitability of the framework for game-based finger rehabilitation through user-experience measures such as Intrinsic Motivation Inventory (IMI), flow experience, and overall gaming experience.

## Introduction

Rehabilitating people with arm disabilities due to old age, neurological disorders or stroke usually require frequent and intense exercise^1,2^. However, lack of motivation is a major hindrance to fast and effective recovery^3,4^. Recently, there is a growing interest in the development of games for purposes outside entertainment such as education^5^, exercise^6^ and memory training^7^. This is because among several other merits such games have been generally associated with increased motivation in the underlying application. Specifically, in the context of rehabilitation, a number of recent studies have demonstrated that exergames (games that are developed to provide physical exercise) can provide the needed motivation and exercise intensity in rehabilitation^8–12^. Rehabilitation games are designed to target specific motor functions that need rehabilitation, whether in a classical or virtual reality (VR) setting^13,14^. For example, in finger rehabilitation for conditions like stroke or arthritis, exercises such as Finger Touch and Thumb Stretch^15^ are commonly used. These clinically established rehabilitation exercises are intentionally designed to engage specific finger muscles and are recognized to enable control of targeted games within a Human–Computer Interaction (HCI) framework. Therefore, since the whole framework usually relies on an end-to-end interaction between patients (in form of exercises) and the targeted exergames (in terms of its control), detecting and interpreting those exercises correctly become crucial.

In arm rehabilitation, especially when focusing on finger rehabilitation, the prescribed exercises tend to be closely aligned with everyday hand gestures such as pinching, grasping, or extending specific fingers. As a result, multiple studies have attempted to adapt general hand gesture recognition approaches for rehabilitative purposes^16–19^. For instance, in^16^, a hand gesture recognition system leveraging sEMG data was employed to enable the real-time control of a 3D exergame. The game’s design incorporated several hand exercises commonly prescribed in clinical settings, thereby allowing individuals with certain disabilities to use low-cost sEMG sensors for interactive rehabilitation. Similarly^17^, relied on well-known hand gesture recognition frameworks that combine computer vision with deep learning, first detecting the user’s hand and then classifying the associated gesture. The recognized gestures were subsequently mapped to input commands in a shooting game, with the overarching goal of supporting wrist rehabilitation. By engaging users in repeated, purposeful movements, these types of games have been shown to boost motivation and adherence.

Moreover, in^20^, the authors harnessed the hand-tracking capabilities of the Microsoft Kinect to create a user-centered rehabilitation game specifically targeting hand and finger exercises. This system not only captured a diverse range of user hand gestures but also provided a structured environment for patients to practice exercises in a playful yet goal-oriented manner. The experiments carried out with various populations demonstrated the feasibility of embedding rehabilitative exercises into exergames and underscored how such an approach can yield positive outcomes across different age groups and levels of disability. Collectively, these studies highlight the versatility and promise of gesture-based exergames as an avenue for delivering targeted, motivating, and potentially more effective rehabilitation experiences.

**Fig. 1:**
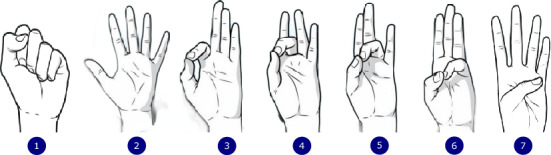
Examples of finger rehabilitation exercise 1) Fist 2) Finger spread 3) Index touch 4) Middle touch 5) Ring touch 6) Pinkie touch and 7) Thumb stretch.

Although the previously mentioned studies have demonstrated the use of exergames for finger or arm rehabilitation, they primarily depend on hand gesture datasets that were not specifically collected for rehabilitative exercises. In other words, most existing systems rely on general-purpose hand gesture datasets intended for tasks like numeric gesture recognition or sign language, which often do not capture the subtle yet crucial motor movements critical for finger rehabilitation. For example, in the context of finger rehabilitation, the seven commonly employed exercises are the Finger touch (representative of 5 classes), Thumb stretch (representative of 1 class), and the Knuckle bend (representative of 1 class) as shown in Fig 1. However, in most cases, all the representative classes in the Finger touch exercise are assumed to be one class in available databases because they were mainly collected for hand number gesture recognition. Furthermore, some of the datasets do not contain certain gestures and would result in either over-exercising or under-exercising some fingers over others, which might lead to undesirable effects. Consequently, this mismatch can diminish the therapeutic impact of exergames, since the recognized gestures may not align well with the clinically prescribed movements that promote recovery. In addition, the global increase in conditions such as stroke and arthritis intensifies the importance of developing tailored datasets. Without such domain-specific data, there is a risk of under-delivering on the specific exercises needed to motivate and guide patients effectively through their rehabilitation programs. Importantly, this challenge is not one of model complexity but of problem alignment: finger rehabilitation gestures are anatomically constrained and not invariant to transformations such as lateral inversion, which are commonly assumed in general-purpose hand gesture recognition. As a result, solutions developed for symbolic or numeric gestures do not directly transfer to rehabilitation settings, even when using powerful deep learning architectures. This motivates a careful rethinking of dataset design, evaluation protocols, and failure analysis specific to rehabilitation-oriented gesture recognition.

Therefore, deviating from the body of previous works, this work proposes a representative hand gesture recognition framework for finger rehabilitation based on transfer learning and a hand-gesture dataset collected specifically for the purpose of finger rehabilitation. The dataset contains 2,800 RGB images representative of seven common finger exercises collected from 14 different subjects under varying environmental conditions. Using this dataset, we developed learning networks by leveraging on transfer learning of off-the-shelf pre-trained VGG16 model in a classical feature extraction scheme as well as fine-tuning to recognize the seven different exercises featured in the dataset. The learning architecture employed in this work is intentionally kept simple and well-established, as the primary goal is not architectural novelty but robust problem formulation, dataset alignment, and system-level validation for rehabilitation-oriented applications. In such settings, interpretability, reproducibility, and deployability are often prioritized over model complexity. The resulting models achieved an accuracy of 82.38% and 85.12% before and after fine-tuning respectively. Furthermore, we provide discussions regarding the underlying challenges of hand-gesture recognition based on datasets collected specifically for finger exercise. To further validate the efficacy of the collected dataset and the trained models, we implemented the best-performing model as control mechanism within a game in a real-world scenario. Specifically, the actions available in the game were assigned to specific gestures in the dataset, enabling participants to control the game while simultaneously performing finger exercises. These types of games fall under the category of exergames^21^ and are associated with increased interest, enjoyment, and exercise intensity in rehabilitation^8,22^. Participant experiences were evaluated using metrics designed to measure flow experience, intrinsic motivation, and overall satisfaction. The analysis of these metrics indicates that the developed models are both lightweight and sufficiently accurate for application in a dedicated exergame aimed at rehabilitation. The main contributions of this work are as follows:

1. A new dataset of hand gestures representative of finger rehabilitation exercises is proposed.
2. Based on the proposed dataset, an off-the-shelf VGG16 model is employed for recognizing hand gestures.
3. A test accuracy of 85.12% is realized after the fine-tuning of the proposed model. Furthermore, the effect of fine-tuning on the model performance is studied.
4. Challenges that arise in recognizing hand gestures that are representative of finger exercises are discussed based on results from Class activation heatmaps.
5. Lastly, we implement the best model as a control mechanism in an exergame and present the results of the user experience using several experience metrics.

The structure of the paper is as follows: Section II describes the data collection procedures and the pre-processing steps involved. Section III provides an overview of transfer learning and its variants. Section IV explains the architecture of the learning model, covering both the feature extraction and fine-tuning processes. Sections V and VI outline the exergame utilized in the study and provide details about the study protocol and participants. Section VII presents the experimental results and discusses the findings. Finally, Section VIII concludes the paper and highlights potential directions for future research.

## Hand gesture dataset description and pre-processing

**Fig. 2:**
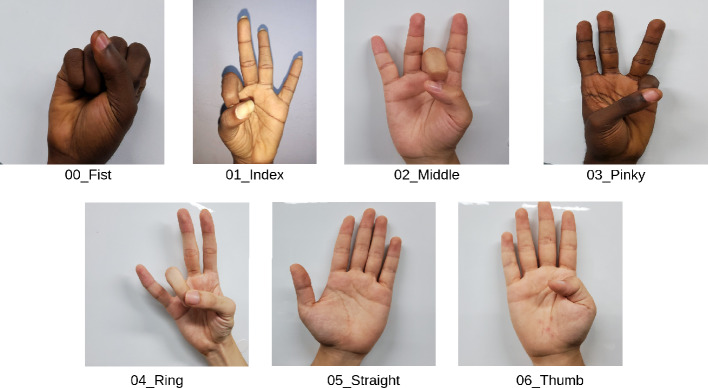
Illustration of the seven (7) hand-gesture classes featured in the dataset.

The dataset comprises RGB images of hand gestures, captured from various directions, backgrounds, and lighting conditions. These images were collected from 14 subjects performing seven distinct finger exercises: Fist, Index Touch, Middle Touch, Ring Touch, Pinkie Touch, Finger Spread, and Thumb Stretch. The subjects were mainly students of Kyungpook National University of which 3 are of Asian descent and 11 were of African descent. The hand exercises featured in the dataset are illustrated in Fig 2. The data for this study was collected with a wide-angle lens camera, having a 12 megapixel resolution and a 26 mm equivalent focal length. The camera used has optical image stabilization and can capture images in RAW or JPEG format, with a color depth of 8 bits. The mean age±standard deviation of the subjects was 27±4 years. A total of 2800 RGB images were collected comprising of 200 images from each subject for all seven classes. For each subject, datasets were collected for both left and right hand. This is to ensure that the learning network will be scalable to either the left or right arm depending on the hand that is to be rehabilitated.

In the context of using the dataset for developing the associated recognition system, the dataset is split into into training and test sets. Specifically, a subject-dependent division of the dataset was employed where data collected from subjects s1, s2, s3, s5, s6, s7, s8, s9 and s10 were used for training and those collected from subjects s11, s12, s13 and s14 were used for testing. The use of subject-based division is important because it ensures that the resulting learning model is not subject-dependent. In order words, the ability of the resulting learning model or recognition system to generalize to hand gestures from unseen subjects during the training process can be tested.

Furthermore, considering that the size of the dataset is not very large, data augmentation is employed. Data augmentation is a collection of image processing techniques that is used to enhance or increase the size and quality of training datasets in order to realize more efficient deep learning models^23^. In the literature, it has been demonstrated from several works^23,24^ that improved performance as well as overcoming the problem of overfitting can be achieved with carefully augmented datasets. To this effect, we select and apply some of the commonly used data augmentation techniques in computer vision as summarised in Table 1. Apart from the advantage of increasing the size of the dataset, our choice of the data augmentation techniques was motivated by the need to ensure that the model is scalable to different hand sizes (through the use of width and height shift), different backgrounds (by applying brightness) and different hand orientations (by applying rotation).

**Table 1:** Parameters for the featured augmentation techniques.

Data Augmentation	Parameter	Value(s)
Rotation	rotation angle	± 20
Brightness	intensity	[0.1, 0.7]
Horizontal flip	logical	True
Height shift	% of the image to shift	0.5
Width shift	% of the image to shift	0.5

## Methods

### Transfer learning

Transfer learning is adopted in this work to address the limited size of rehabilitation-oriented hand gesture datasets, where training deep networks from scratch is prone to overfitting. Specifically, a model pre-trained on ImageNet is repurposed to learn discriminative representations of finger-level hand configurations under varying backgrounds and lighting conditions. For example, learning how to play the Piano may help facilitate learning the acoustic guitar faster and more easily. According to^25,26^, given a source domain $D_{S}$ and its associated learning task $\tau _{S}$, as well as a target domain $D_{T}$ and its associated learning task $\tau _{T}$, transfer learning is an approach that aims to improve the learning of the target predictive function $f_{T}(\cdot )$ (an objective predictive function, learned from the training data) in $D_{T}$ using the knowledge in $D_{S}$ and $\tau _{S}$, where $D_{S} \ne D_{T}$, or $\tau _{S} \ne T_{T}$. In other words, features and weights from a successful source domain and its tasks are utilize to improves the performance of the target task. Depending on the target task, one of the main reasons why transfer learning techniques are deployed is to take advantage of accurate learning models realized based on large training datasets to overcome the limitations of the target task in terms of small datasets and or limited labels^27^. Although there are several techniques or paradigms regarding how and what knowledge to transfer in transfer learning^26^. The two most common strategies are feature extraction and fine tuning. Similarly, in this work, we employ feature extraction and fine tuning based on an off-the-shelf pre-trained Model.

**Fig. 3:**
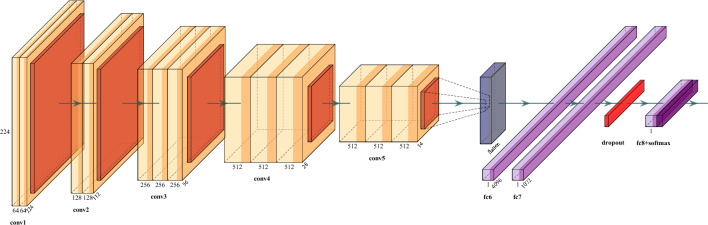
Architecture of the learning model.

### Feature extraction

The success of any leaning network usually depends on its ability to extract meaningful features that are critical its associated task^28^. Feature extraction under the transfer learning scheme generally involves utilizing the representations learned by the source task to facilitate the extraction of meaningful features from new samples for the target task^29^. In other words, the weighted layers of the source task are frozen (weights are not updated) but only used to extract features during training with new data for the target task. In terms of implementation, a new classifier that is trained from scratch, is added on top of the pretrained model (on the source task) so that its feature maps learned previously for the source task can be repurposed for the target task. In this configuration, all convolutional layers of the VGG16 backbone are frozen, allowing the network to leverage generic visual features while evaluating their suitability for distinguishing rehabilitation-specific finger exercises.

### Fine tuning

In fine tuning, instead of relying solely on fixed representations learned from the source task, a subset of higher-level layers in the pretrained base network is unfrozen and jointly optimized together with the newly added classifier layers. This strategy allows task-specific adaptation of abstract feature representations while retaining the general visual descriptors learned in earlier layers^30,31^.

In this work, only the last four convolutional layers of the VGG16 backbone are unfrozen during training. These layers capture higher-order semantic information such as finger configuration and relative digit positioning, which are critical for distinguishing subtle inter-finger rehabilitation gestures. Lower-level convolutional layers, which primarily encode generic edge, texture, and spatial features, are kept frozen to preserve stable visual representations and reduce the risk of overfitting given the limited size of the rehabilitation-oriented dataset. This partial fine-tuning strategy enables a controlled comparison against pure feature extraction, allowing us to evaluate the extent to which task-specific adaptation improves recognition performance without introducing confounding effects from full network retraining.

### Learning model architecture

Considering that the two TL schemes employed in this work requires an off-the-shelf pre-trained base mode with good performance on its associated source task, some high performing networks for computer vision tasks that comes to mind includes VGG-16^32^, VGG-19^32^, Inception V3^33^ and ResNet-50^34^ etc. For the purpose of this work, VGG16 is chosen as the base model. Our choice of VGG16 is motivated by its good performance, ease of implementation and its adoption for most TL tasks^35^. Furthermore, while more recent architectures exist, the use of a well-understood transfer learning framework allows a focused investigation of rehabilitation-specific challenges without confounding effects from architectural complexity.

In terms of implementation, the fully connected layer of the VGG16 base model is removed and replaced with a new classifier as shown in Fig. 3. In the classifier, the output of the VGG block is flattened and passed to a series of two dense layers with 4096 and 1072 neurons respectively that are activated using ReLU. Furthermore, a dropout layer is appended prior to the final output layer with Softmax activation function to prevent over-fitting.

Based on the model shown in Fig.3, two different models are trained based on feature extraction and fine-tuning respectively. The models have the same architecture but differ slightly in the number of layers of the base VGG16 network that are used for training. Specifically, in the first model (based on feature extraction) the value of the fine-tuning parameter which dictates the number of layers in the convolutional base that are switched from non-trainable to trainable is set to zero (0) implying that new model described in Fig. 3 will be bootstrapped onto the pre-trained VGG base. During the training process, weights of only the newly added model are updated while keeping the pre-trained weights of the VGG block as it is. In the second model, a fine-tuning value of 4 is used implying that the last 4 layers of the base VGG 16 base network are unfrozen during training. Consequently, the weights of these layers are updated in conjecture with the weights of the newly added top model.

## Rehabilitation exergame

**Fig. 4:**
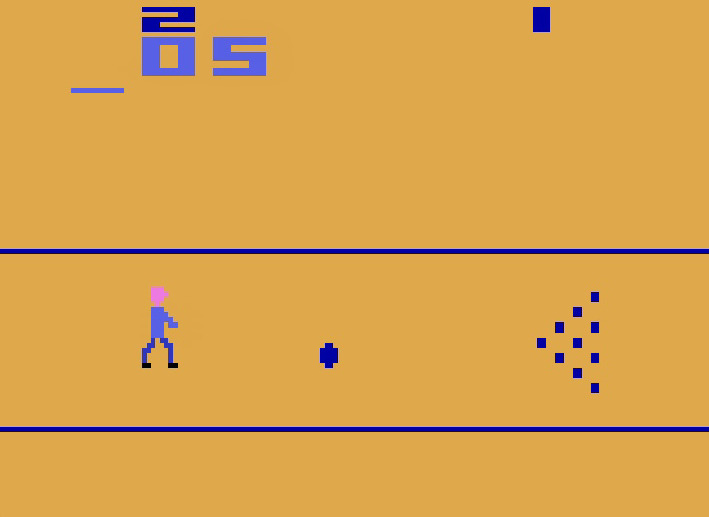
A still-frame from the rehabilitation exergame.

In this study, to demonstrate the robustness of the developed dataset and the associated gesture recognition the trained model are deployed in a real-world scenario. The hand rehabilitation exergame used for this study is the Bowling Atari environment available in the Gymnasium framework as shown in Fig. 4. In Bowling, the objective is to score as many points as possible over 10 frames, with two tries per frame. Knocking down all pins on the first attempt within a frame is termed a “strike,” which awards 10 points plus the total points from the next two rolls. If all pins are knocked down on the second attempt, it is termed a “spare,” awarding 10 points plus the points from the subsequent roll. When not all pins are knocked down after two attempts, the frame is considered “open,” and the score is the total number of pins knocked down in that frame. The maximum attainable score in bowling is 300, achieved by rolling 12 consecutive strikes.

The six actions available in this game is mapped to six gestures in the dataset. The actions and their associated gestures are summarised in Table 2. In this study, one participant controls the player and actions by performing actions which is captured by a 1280x720 pixel camera at 30 frames per second. Each frame is consequently passed to a pre-processing module which identifies the primary hand (hand that is performing the gesture) in the frame (in case of occurrence of multiple hands). The extracted image of the hand is then passed on to the trained gesture recognition module, the output of which is mapped to the action space of the game. The choice of this game for this study lies in its game design which imitates real-world rules hence providing an authentic experience but incorporates certain unique features that renders it appropriate as an exergame. Contrary to the real-world version of bowling, this game allows the player to modify the trajectory of the bowl after being thrown, albeit only once. Once thrown, the bowl can be either moved upwards or downwards using the actions with IDs 2 and 3, which also double as actions to move the player up and down respectively. Actions 4 and 5 allow the throwing of the ball from a stationary position of the player upwards and downwards respectively. This schematic of actions help the user exercise their motor control (performing a gesture properly as to avoid confusion with another gesture) as well as motor planning (planning and executing the movement of the ball at the right time), keeping the user engaged with the exergame. Furthermore, this game is employed as the exergame motivated by the framework that ensures the active usage of all finger exercises considered in the recognition system. At the same time, the relatively slower pace and easy to understand rules of the game ensures that the user is not overwhelmed by the decisions that need to be made in the game.

**Table 2:** Actions available in the Bowling environment and the corresponding gestures.

Action ID	Action	Gesture
0	No Action	06_Thumb
1	Fire	05_Straight
2	Move Player Up	01_Index
3	Move Player Down	03_Pinky
4	UPFIRE	02_Middle
5	DOWNFIRE	04_Ring

## Study protocol and participants

In this work, the study employed the use of Bowling from the Gymnasium framework and reformulated it as an exergame. As discussed in Section 4, this was realized by mapping the in-game actions available to the gestures present in the proposed dataset. The participants were asked to engage themselves in the exergame and their experience as well as the effect were quantified using three self-report questionnaires: Flow Experience Metric (FEM), Intrinsic Motivation Metric (IMI) and Overall Experience Metric. FEM is used to measure the psychological state of flow, characterized by by complete immersion and involvement in an activity, often leading to a sense of enjoyment and intrinsic motivation. Metrics for assessing flow typically involve evaluating factors such as concentration, enjoyment, control, and challenge-skill balance^36^. To quantify the flow state, the questionare used by^37^ was employed which contains 8 questions with five outcome variables. Similarly, the participants also completed the Intrinsic Motivation Inventory (IMI) questionnaire, which consisted of eight statements rated on a Likert scale from 1 to 7. The shortened version of this questionare as proposed in^38^ measures four scales-interest/enjoyment, effort/importance, perceived competence, and pressure/tension-each with two items, yielding scores between 2 and 14 per scale. Six questions forming the part of the Overall Experience Metric (OEM)^17^ was also employed to measure the user’s interest and innate sense of accomplishment while playing the game. Furthermore, the OEM questionnaire also measures the occurrence of any pain during the game-play which signifies gestures that activate optimal regions of their hand enabling quick rehabilitation.

The study was conducted on 15 (13 Male and 2 Female) participants between the ages of 21 and 39 with a mean age of 27.2 ± 4.88. Among the participants included individuals with dominant right hand as well as those with a dominant left hand. For the study, the participants played the exergame in two sessions each, spaced 2 minutes apart. Each session lasted for 5 minutes during which they attempted to score the maximum points according to the rules described in Section 4. Prior to the starting of the game, the participants were allowed to practice interacting with the exergame using the gestures in the dataset. This helps the participants become familiar with mapping gestures to specific in-game actions, making the subsequent two sessions more streamlined. After finishing the second 5 minute session, the participants were presented with the questionnaire measuring the FEM, IMI and the Overall Experience metrics.

## Experimental results and discussions

### Performance evaluation of hand gesture recognition model

In this Section, the experimental setup as well as the results of the experiments conducted in this work to evaluate the performance of the leading networks based on the hand gesture dataset are presented. All the experiments performed in this work were conducted in Python with TensorFlow installed on a computer with an NVIDIA TITAN RTX GPU of a compute capability of 7.5 and a base clock speed of 1350MHz. In terms of the training the learning networks, the hyperparmaters employed as presented in Table 3 were obtained based several rounds of trial and error.

**Table 3:** Training parameters.

Parameter	Feature Extraction	Fine Tuning
Dense layer activation	ReLU	ReLU
Output layer activation	softmax	softmax
Loss function	categorical cross entropy	categorical cross entropy
Optimizer	Adam	Adam
Learning Rate	0.001	0.0001
Epochs	100	100
Unfrozen layers	4	0

The performance of the classifiers are evaluated based on their precision, recall, and F1-score during inference on the test data. Generally, Precision is the ratio of the positive class predictions that were actually correct and can be expressed mathematically as:

1 $$\begin{aligned} Precision = \frac{TP}{TP+FP} \end{aligned}$$

where TP is the total number of True positives and FP is the total number of False positives. Recall on the other hand is the ratio of the actual positive class samples that were identified by the model which is expressed mathematically as:

2 $$\begin{aligned} Recall = \frac{TP}{TP+FN} \end{aligned}$$

where FN is the total number of False negatives. F1-score is a measure of accuracy of the models on correctly classifying the different hand gestures and is calculated as follows:

3 $$\begin{aligned} F1\hspace{0.1cm}score = 2\times \frac{P\times R}{P+R} \end{aligned}$$

where P and R denotes the precision and recall respectively. In a multi-class scenario as in this work, the precision and recall are computed in a class-wise fashion. Furthermore, an early stopping condition is introduced during the training stage such that the training is ended if no improvement in the validation loss is observed after 30 consecutive epochs.

In Table 4, the class-wise precision, recall and F1-score as well as their average values for the feature extraction-based and the fine-tuned model respectively are presented.

**Table 4:** Class-wise and average Precision, Recall and F1-scores for the model without fine tuning.

Class	Feature Extraction			Fine Tuning		
	Precision	Recall	F1-Score	Precision	Recall	F1-Score
00_Fist	1.000	0.883	0.931	1.000	0.950	0.974
01_Index	0.771	0.958	0.855	0.686	0.983	0.808
02_Middle	0.699	0.833	0.760	0.715	0.775	0.744
03_Pinky	0.905	0.875	0.889	0.947	0.891	0.918
04_Ring	0.717	0.591	0.648	0.888	0.600	0.716
05_Straight	0.816	1.000	0.898	0.867	0.983	0.921
06_Thumb	0.937	0.625	0.750	0.989	0.775	0.869
Average	0.835	0.823	0.818	0.870	0.851	0.850

**Fig. 5:**
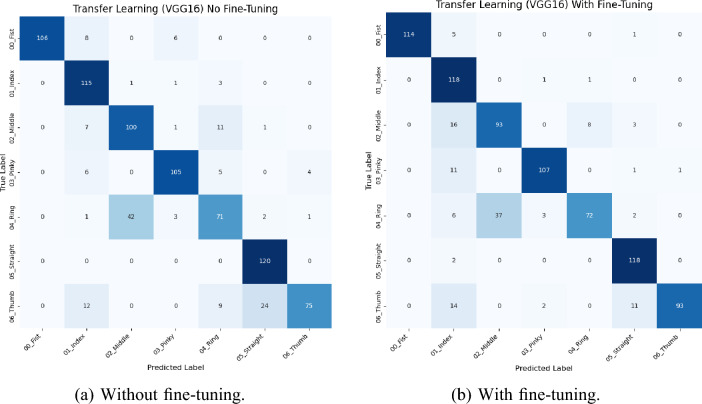
Confusion matrices for the model before and after fine-tuning.

It can be observed that a percentage improvement of 4.19%, 3.40%, and 3.91% is achieved in the precision, recall, and F1-score respectively following the fine-tuning of the feature extraction model. Furthermore, to present an in-depth insight into the results, the confusion matrices are presented in Fig. 5a and b for the model without and with fine tuning respectively. From a brief overview of the confusion matrices, an overall improvement in gesture recognition accuracy is observed from 82.38% to 85.12% after the incorporation of fine-tuning into the training process. An example of the impact of fine tuning is observed by a 24 % increase in the accuracy for the class of “06_Thumb”.

**Fig. 6:**
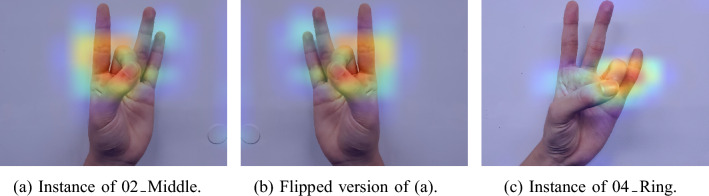
Class activation heatmaps showing the features used by the model to distinguish the two classes of “02_Middle” and “04_Ring” demonstrating the conflict.

Furthermore, in the confusion matrices for both models, it can be seen that the two classes that return the highest level of misclassification with each other are the classes of “02_Middle” and “04_Ring”. Intuitively it can be argued that this is due to the fact that the dataset was collected from both left and right hand as well as the horizontal flipping during the augmentation process which are aimed at ensuring that the resulting model would be robust to gestures from both hands. This is because flipping the “02_Middle” class results into an image that is visually similar to an image from the “04_Ring” class and the only distinguishing feature between the two classes are the location of the thumb which is at a distance from the other four fingers. To validate this observation, we present the Class Activation Map (CAM) for the learning network to investigate the features or regions in the images that inform the decision of the classifier. In Fig. 6, the class activation heat maps of a sample image from the class “02_Middle” (Fig. 6a) and its augmented version based on the horizontal flip operation (fig. 6b) as well as that of an image from the “04_Ring” class (Fig. 6c) are presented. It can be observed from the images that the flipped image is quite similar in terms of the arrangements of the fingers to the image of an instance from “04_Ring” depicted in Fig. 6c. Both these images contain three fingers that are fully visible and the folded middle finger (in (fig. 6b)) is visually similar to the folded ring finger (in (fig. 6c)). This anatomical characteristic induces misclassification as seen from the confusion matrices. Although the orientation of the thumb finger is a discernible difference to the human eye, it accounts for just a small part of the image and other features predominate the thumb position. Such artifact is absent from other hand gesture recognition datasets as their primary goal is usually to recognize alphabets or numbers. Hence models developed based on those datasets would not be efficient for classifying finger exercises that are invariant to lateral inversion. In spite of these challenges, fine-tuning of the VGG16 model helps to reduce the misclassification error associated with the “02_Middle” and “04_Ring” classes. Specifically, a percentage improvement of 23.8% and 2.28% is achieved in the precision for the “02_Middle” and “04_Ring” respectively.

### User study results and discussions

In this study, we comprehensively evaluated the user experience of participants engaging with a virtual bowling exergame designed to promote hand exercise and injury recovery through the use of hand gestures. The gesture recognition model, trained on a novel dataset, was assessed using various user experience metrics to gauge its effectiveness and participant satisfaction. It is worth noting that the effectiveness of the proposed framework is evaluated at the system level, where reliable interaction and user engagement are more critical than marginal gains from architectural complexity.

The demographic distribution of participants spans a wide age range with a slightly higher representation of males. This diversity is important as it demonstrates the game’s broad appeal and applicability across different age groups. Understanding the demographic engagement helps in customizing and refining the game to cater to the needs of various user segments, thereby enhancing its overall effectiveness and user satisfaction. The broad age range also suggests that the game is accessible and engaging for both younger and older participants, which is crucial for ensuring wide adoption and use in therapeutic settings.

**Fig. 7:**
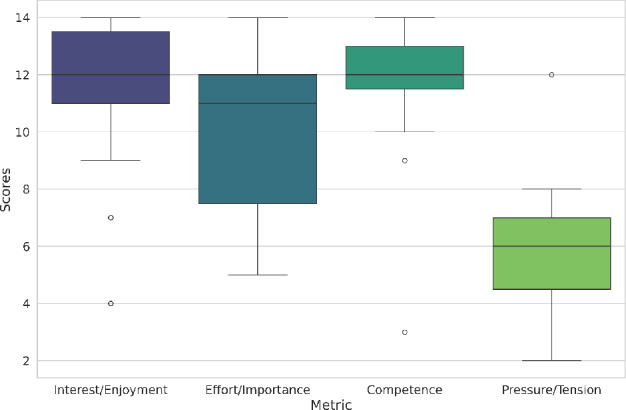
Distribution of Intrinsic Motivation Inventory scores. The x-axis represents the different metrics used to calculate the IMI scores while the y-axis itself represents the IMI score for each metric. The outliers in the data are denoted by the circles in the plot.

Following the demographic overview, the Intrinsic Motivation Inventory (IMI) scores, presented in Figure 7, provide deeper insights into the motivational aspects of the game. The scores across four subscales-Interest/Enjoyment, Effort/Importance, Competence, and Pressure/Tension-reveal that participants generally found the game enjoyable and felt a sense of mastery. High scores in Interest/Enjoyment and Competence indicate that the game is not only engaging but also fosters a sense of skill development, which can enhance the likelihood of continued use and adherence to the exercise routine^39^. The Effort/Importance scores, while lower than the enjoyment and competence metrics, still highlight that participants recognized the effort required by the game, underscoring the balance between challenge and skill. Importantly, the low Pressure/Tension scores suggest that the game environment is not stress-inducing, which is essential for maintaining a positive user experience in a therapeutic context. This level of perceived effort is expected and appropriate, as the gestures implemented in the system correspond to clinically prescribed rehabilitation exercises that deliberately engage specific finger muscles rather than prioritizing ease.

**Fig. 8:**
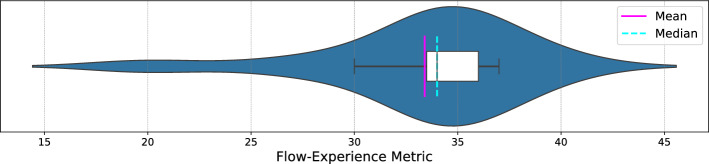
Distribution of the scores of the participants on the Flow Experience Metric. Higher Flow scores correlate to more immersion in the activity and a better user experience.

Building on the motivational aspects, the Flow Experience Metric, depicted in Figure 8, provides a detailed view of the participants’ immersion levels during gameplay. The Violin plot, which combines box plot and density plot features, indicates that the majority of participants experienced a high level of immersion, with scores clustering around the mean and median values. This high flow state is indicative of deep engagement and suggests that the game successfully captures and maintains participant attention, which is crucial for both enjoyment and therapeutic effectiveness. A high flow experience typically correlates with increased motivation and a greater likelihood of adherence to the exercise routine, which is beneficial for recovery purposes. The flow metric complements the motivational scores by demonstrating that the game is not only enjoyable but also deeply engaging, enhancing the overall user experience.

**Fig. 9:**
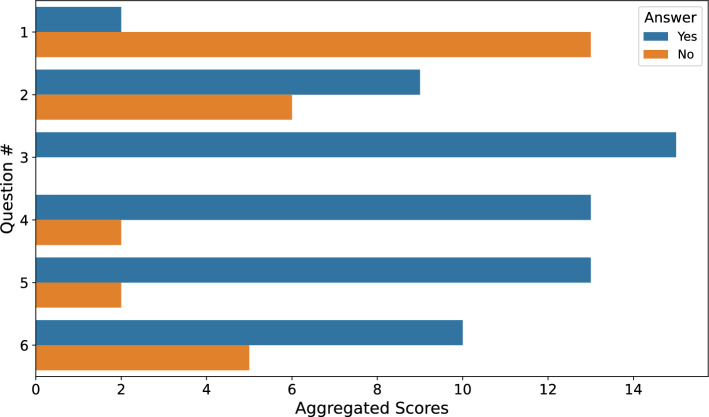
Histogram depicting the responses of the participants to the 6 questions under Overall Experience Metric.

Finally, the Overall Experience Metric, as illustrated in Figure 9, provides a detailed account of participant responses to six key questions designed to capture their overall impressions of the game. These questions were: “Do you find the game difficult to play?”, “Did you experience pain while playing the game?”, “Did you find the game effective?”, “Did you have good control over the game?”, “Did you find the game interesting?”, and “Do you think you will use the exergame on a daily basis?”. The histogram reveals a mixed set of responses, with notable observations. For instance, the higher proportion of “No” answers to the question about difficulty indicates that most participants did not find the game overly challenging, which is essential for maintaining accessibility and encouraging continued use. Similarly, the low number of “Yes” responses to experiencing pain suggests that the game is physically comfortable to play, aligning well with its therapeutic objectives. The high number of affirmative responses regarding the game’s effectiveness and interest level underscores its potential as an engaging and beneficial tool for hand exercise and recovery. However, the mixed responses to daily use highlight an area for potential improvement, indicating that while the game is effective and engaging, further enhancements might be needed to fully integrate it into participants’ daily routines.

Importantly, the purpose of this user study is not only to assess subjective enjoyment, but also to evaluate whether a gesture recognition system with moderate frame-level errors can remain effective at the system level when embedded in a rehabilitation-oriented human–computer interaction loop. This distinction is particularly relevant for rehabilitation applications, where continuous control and user perception are often more critical than isolated instance-level classification accuracy.

The detailed analysis of user experience metrics indicates that the Bowling exergame, powered by the gesture recognition model from our proposed dataset, provides a highly engaging and positive experience for participants. The high levels of interest and perceived competence, coupled with low pressure and tension, suggest that the game is well-suited for its intended purpose of facilitating hand exercises and aiding in injury recovery. Furthermore, it can be observed from the user experience study that although the underlying classification model has a misclassification rate of 14.88%, it does not affect the overall system performance. This is because the control of the exergame is continuous and not instance-based, hence, the effect of the misclassification is not noticeable. In other words, the continuous hand exercise recognition makes the effect of frame or instance-based missclassification negligible.

## Limitations of the current study

The approach presented here is intended as a foundational concept for integrating gesture recognition into exergames designed for finger rehabilitation, yet several limitations point toward areas requiring further refinement. In particular, the proposed framework relies on vision-based hand gesture recognition and therefore assumes the presence of at least partial hand and finger mobility. As such, it is not intended for rehabilitation scenarios involving complete hand loss or extremely severe muscle weakness, where meaningful visual gestures cannot be formed and alternative modalities such as sEMG-based interfaces, prosthetic systems, or therapist-assisted interventions are more appropriate.

The participant demographic in this preliminary study consists primarily of healthy and relatively young individuals, and therefore does not fully capture the spectrum of movement patterns observed in clinical populations. Although data augmentation techniques such as rotations, shifts, and brightness adjustments can partially simulate variability in hand appearance, they cannot replicate the reduced amplitude, irregular motion, or compensatory strategies often exhibited by older adults or individuals recovering from conditions such as stroke or arthritis. This limitation reflects a broader sim-to-real challenge when transferring systems validated in controlled environments to real-world rehabilitation settings. Nevertheless, similar to several prior works in the literature^37,39,40^, the use of healthy participants is appropriate at this stage, as the present study is not intended as a clinical trial focused on rehabilitation outcomes. Instead, the proposed framework establishes an intuitive end-to-end pipeline for finger-based rehabilitation exergames and serves as a foundation for future studies involving impaired populations and clinically validated therapeutic evaluation.

Additionally, no formal clinical validation involving therapists or actual rehabilitation patients has yet been performed. Such collaboration is crucial for determining the system’s feasibility, therapeutic impact, and alignment with real-world clinical workflows, including aspects such as cost, training requirements, and user onboarding. While the accuracy scores achieved thus far indicate that the system is suitable as a proof-of-concept, misclassifications in a therapeutic context could diminish motivation and potentially impede rehabilitation goals. Consequently, bridging the “sim-to-real” gap necessitates additional measures to ensure that the exergame’s gesture recognition remains both reliable and user-friendly when deployed in actual therapy sessions.

## Conclusion and future works

In this paper, a novel hand gesture dataset comprising seven common hand exercises for finger rehabilitation through exergames was introduced. Utilizing this dataset, a hand gesture recognition model leveraging transfer learning from the pretrained VGG16 model was developed, achieving classification accuracies of 82.38% (feature extraction) and 85.12% (fine-tuning) on the test dataset. Notably, the highest misclassification rates were observed in the middle and ring classes due to similar regions of interest being activated for flipped versions of these gestures, underscoring the need for datasets specifically aligned with rehabilitation exercises where invariance to lateral inversion is crucial. By curating and validating a dataset oriented toward finger rehabilitation, we addressed the previously stated challenge of ensuring that recognized gestures correspond closely to clinical or therapeutic requirements.

To assess the practical application of the model, a user experience study was conducted using a Bowling exergame controlled by hand gestures. Participants, spanning a diverse age range, provided high scores in Interest/Enjoyment and Competence, along with low scores in Pressure/Tension. These results indicate that the game is both engaging and effective, which is essential for maintaining participant motivation and ensuring therapeutic benefits. Furthermore, the Flow Experience Metric showed high levels of immersion among participants, underscoring the game’s ability to capture and maintain user attention, which is crucial for rehabilitation success. Additionally, the Overall Experience Metric revealed that participants generally found the game easy to play, pain-free, interesting, and effective. However, mixed responses regarding the likelihood of daily use suggest that there is room for further refinements to fully integrate the game into users’ daily routines, which could enhance the game’s long-term adherence and therapeutic impact.

While this work adopts a well-established transfer learning framework, it is intended as a foundational step toward rehabilitation-oriented gesture recognition rather than an exploration of architectural novelty. The proposed dataset, analysis, and system-level evaluation provide a principled baseline upon which more advanced models such as temporal architectures, attention-based networks, or multimodal approaches can be systematically explored in future work.

Accordingly, future work will focus on expanding the dataset to include a more diverse demographic and age range, with particular emphasis on elderly and impaired populations who are more likely to require rehabilitative interventions. Incorporating participants with motor impairments into both data collection and user experience evaluation will further strengthen the robustness and practical applicability of the proposed framework. By refining these aspects, we aim to reduce misclassification rates, promote sustained user engagement, and reinforce the effectiveness of exergames as a compelling platform for finger rehabilitation.

## Data Availability

The datasets generated during and/or analyzed during the current study are available from the corresponding author upon reasonable request.

## References

[1] MacKay-Lyons, M. et al. Aerobic exercise recommendations to optimize best practices in care after stroke: Aerobics 2019 update. *Phys. Ther.* **100**(1), 149–156 (2020). 31596465 10.1093/ptj/pzz153 PMC8204880

[2] Gittler, M. & Davis, A. M. Guidelines for adult stroke rehabilitation and recovery. *JAMA* **319**(8), 820–821 (2018). 29486016 10.1001/jama.2017.22036

[3] Gillison, F. B., Rouse, P., Standage, M., Sebire, S. J. & Ryan, R. M. A meta-analysis of techniques to promote motivation for health behaviour change from a self-determination theory perspective. *Health Psychol. Rev.* **13**(1), 110–130 (2019). 30295176 10.1080/17437199.2018.1534071

[4] Brand, R. & Cheval, B. Theories to explain exercise motivation and physical inactivity: Ways of expanding our current theoretical perspective. Frontiers in Psychology 10 (2019). 10.3389/fpsyg.2019.01147 PMC6536603 31164856

[5] Bontchev, B. & Vassileva, D. Affect-based adaptation of an applied video game for educational purposes. *Interactive Technology and Smart Education* **14**, 31–49 (2017).

[6] Vera-Ponce, V. J., Ballena-Caicedo, J., Zuzunaga-Montoya, F. E. & Gutierrez De Carrillo, C. I. Effectiveness of active video games for promoting physical activity: An umbrella review. *Front. Sports Act. Living* **7**, 1706145 (2025). 41333800 10.3389/fspor.2025.1706145 PMC12666624

[7] Yaqian, Z. & Wooi-Boon, G. Personalized task difficulty adaptation based on reinforcement learning. *User Model. User Adapt. Interact.* **31**(4), 753–784 (2021).

[8] Goršič, M., Cikajlo, I. & Novak, D. Competitive and cooperative arm rehabilitation games played by a patient and unimpaired person: Effects on motivation and exercise intensity. *J. Neuroeng. Rehabil.* **14**(1), 23 (2017). 28330504 10.1186/s12984-017-0231-4 PMC5363008

[9] Moss, T. et al. Intergroup competition in exergames: Further tests of the Köhler effect. *Games Health J.* **7**(4), 240–245 (2018). 29958003 10.1089/g4h.2017.0122

[10] Gorsic, M., Cikajlo, I. & Novak, D. Competitive and cooperative arm rehabilitation games played by a patient and unimpaired person: Effects on motivation and exercise intensity. *J. Neuroeng. Rehabil.*10.1186/s12984-017-0231-4 (2017). 28330504 10.1186/s12984-017-0231-4 PMC5363008

[11] Chen, J., Zhao, S., Meng, H., Cheng, X. & Tan, W. An interactive game for rehabilitation based on real-time hand gesture recognition. *Front. Physiol.* **13**, 1028907 (2022). 36388091 10.3389/fphys.2022.1028907 PMC9643738

[12] Bonnechère, B. Serious Games in Rehabilitation, pp. 41–109. Springer, Cham (2018). 10.1007/978-3-319-66122-3_4.

[13] Shah, S.H.H., Karlsen, A.S.T., Solberg, M. & Hameed, I.A. A social vr-based collaborative exergame for rehabilitation: codesign, development and user study. Virtual Reality, 1–18 (2022). 10.1007/s10055-022-00721-8 PMC9702607 36465891

[14] Trombetta, M. et al. Motion rehab ave 3d: A vr-based exergame for post-stroke rehabilitation. *Comput. Methods Programs Biomed.* **151**, 15–20. 10.1016/j.cmpb.2017.08.008 (2017). 28946996 10.1016/j.cmpb.2017.08.008

[15] Migala, J. 7 Easy Hand Exercises to Prevent Arthritis. https://www.aarp.org/health/conditions-treatments/info-2021/exercises-prevent-arthritis.html.

[16] Nasri, N., Orts-Escolano, S. & Cazorla, M. An semg-controlled 3d game for rehabilitation therapies: Real-time time hand gesture recognition using deep learning techniques. *Sensors*10.3390/s20226451 (2020). 33198083 10.3390/s20226451 PMC7696342

[17] Farahanipad, F., Nambiappan, H.R., Jaiswal, A., Kyrarini, M. & Makedon, F.: Hand-reha: Dynamic hand gesture recognition for game-based wrist rehabilitation. In: Proceedings of the 13th ACM International Conference on PErvasive Technologies Related to Assistive Environments. PETRA ’20. Association for Computing Machinery, New York, NY, USA (2020). 10.1145/3389189.3392608.

[18] Khalaf, A. S., Alharthi, S. A., Alshehri, A., Dolgov, I. & Toups, Z. O. A comparative study of hand-gesture recognition devices for games. In *Human-Computer Interaction. Multimodal and Natural Interaction* (ed. Kurosu, M.) 57–76 (Springer, 2020).

[19] Silva, J. A., Silva, M. F., Oliveira, H. P. & Rocha, C. D. Developing a serious video game to engage the upper limb post-stroke rehabilitation. *Appl. Sci.* **15**(15), 8240 (2025).

[20] Proffitt, R., Sevick, M., Chang, C.-Y. & Lange, B. User-centered design of a controller-free game for hand rehabilitation. *Games Health J.* **4**(4), 259–264 (2015). 26182212 10.1089/g4h.2014.0122 PMC4601630

[21] Zhuang, F. et al. A comprehensive survey on transfer learning. *Proceedings of the IEEE* **109**(1), 43–76 (2020).

[22] Alimanova, M., Borambayeva, S., Kozhamzharova, D., Kurmangaiyeva, N., Ospanova, D., Tyulepberdinova, G., Gaziz, G. & Kassenkhan, A. Gamification of hand rehabilitation process using virtual reality tools: Using leap motion for hand rehabilitation. In: 2017 First IEEE International Conference on Robotic Computing (IRC), pp. 336–339 (2017). IEEE.

[23] Shorten, C. & Khoshgoftaar, T. M. A survey on image data augmentation for deep learning. *J. Big Data* **6**, 1–48 (2019). 10.1186/s40537-021-00492-0 PMC8287113 34306963

[24] Adedigba, A. P., Adeshina, S. A., Aina, O. E. & Aibinu, A. M. Optimal hyperparameter selection of deep learning models for covid-19 chest x-ray classification. *Intell.-Based Med.* **5**, 100034. 10.1016/j.ibmed.2021.100034 (2021). 33899036 10.1016/j.ibmed.2021.100034 PMC8057926

[25] Kornblith, S., Shlens, J. & Le, Q.V. Do better imagenet models transfer better? In: Proceedings of the IEEE/CVF Conference on Computer Vision and Pattern Recognition, pp. 2661–2671 (2019).

[26] Weiss, K. R., Khoshgoftaar, T. M. & Wang, D. A survey of transfer learning. *J. Big Data* **3**, 1–40 (2016).

[27] Ge, W. & Yu, Y. Borrowing treasures from the wealthy: Deep transfer learning through selective joint fine-tuning. In: 2017 IEEE Conference on Computer Vision and Pattern Recognition (CVPR), pp. 10–19 (2017). 10.1109/CVPR.2017.9.

[28] Dara, S. & Tumma, P. Feature extraction by using deep learning: A survey. In: 2018 Second International Conference on Electronics, Communication and Aerospace Technology (ICECA), pp. 1795–1801 (2018). 10.1109/ICECA.2018.8474912.

[29] Chen, L.-C., Zhu, Y., Papandreou, G., Schroff, F. & Adam, H. Encoder-decoder with atrous separable convolution for semantic image segmentation. In: Proceedings of the European Conference on Computer Vision (ECCV), pp. 801–818 (2018).

[30] Long, J., Shelhamer, E. & Darrell, T. Fully convolutional networks for semantic segmentation. In: 2015 IEEE Conference on Computer Vision and Pattern Recognition (CVPR), pp. 3431–3440 (2015). 10.1109/CVPR.2015.7298965. 10.1109/TPAMI.2016.2572683 27244717

[31] Xu, Y. et al. Large scale tissue histopathology image classification, segmentation, and visualization via deep convolutional activation features. *BMC Bioinformatics* **18**(1), 281 (2017). 28549410 10.1186/s12859-017-1685-x PMC5446756

[32] Simonyan, K. & Zisserman, A. Very deep convolutional networks for large-scale image recognition. ICLR abs/1409.1556 (2016).

[33] Szegedy, C., Liu, W., Jia, Y., Sermanet, P., Reed, S., Anguelov, D., Erhan, D., Vanhoucke, V. & Rabinovich, A. Going deeper with convolutions. In: 2015 IEEE Conference on Computer Vision and Pattern Recognition (CVPR), pp. 1–9 (2015).

[34] He, K., Zhang, X., Ren, S. & Sun, J. Deep residual learning for image recognition. 2016 IEEE Conference on Computer Vision and Pattern Recognition (CVPR), 770–778 (2016).

[35] Kaur, S. et al. Transfer learning-based automatic hurricane damage detection using satellite images. *Electronics*10.3390/electronics11091448 (2022).

[36] Michailidis, L. & Barcias, J.L. Hold me tight: Towards the detection of the flow experience using a commercial video game controller. Computers in Human Behavior Reports, 100900 (2025).

[37] Darzi, A., McCrea, S. M. & Novak, D. User experience with dynamic difficulty adjustment methods for an affective exergame: Comparative laboratory-based study. *JMIR Serious Games* **9**(2), 25771 (2021). 10.2196/25771 PMC8204235 34057423

[38] Goršič, M., Darzi, A. & Novak, D. Comparison of two difficulty adaptation strategies for competitive arm rehabilitation exercises. In: 2017 International Conference on Rehabilitation Robotics (ICORR), pp. 640–645 (2017). 10.1109/ICORR.2017.8009320. 10.1109/ICORR.2017.8009320 PMC5669049 28813892

[39] Ajani, O. S. & Mallipeddi, R. Pareto-based dynamic difficulty adjustment of a competitive exergame for arm rehabilitation. *Int. J. Hum. Comput. Stud.* **178**, 103100 (2023).

[40] Goršič, M., Darzi, A. & Novak, D. Comparison of two difficulty adaptation strategies for competitive arm rehabilitation exercises. In: 2017 International Conference on Rehabilitation Robotics (ICORR), pp. 640–645 (2017). 10.1109/ICORR.2017.8009320 PMC5669049 28813892

